# Soluble guanylyl cyclase mediates concomitant coronary vasodilator and positive inotropic actions of the HNO donor Angeli's salt in the intact rat heart

**DOI:** 10.1186/2050-6511-14-S1-P57

**Published:** 2013-08-29

**Authors:** Rebecca Ritchie, Kai Yee Chin, Chengxue Qin, Nga Cao, Barbara Kemp-Harper, Owen Woodman

**Affiliations:** 1 Heart Failure Pharmacology, Baker IDI Heart & Diabetes Institute, Melbourne, Vic, 8008, Australia; 2 Department of Medicine, Monash University, Clayton, Vic 3800, Australia; 3 School of Medical Sciences, RMIT University, Bundoora, Vic 3083, Australia; 4 Department of Pharmacology, Monash University, Clayton, Vic 3800, Australia

## Background

The NO redox sibling nitroxyl (HNO) elicits soluble guanylyl cyclase (sGC)-dependent vasodilatation. HNO has high reactivity with thiols (unlike NO), which is attributed with HNO-enhanced left ventricular (LV) function. The present study tested the hypothesis that the concomitant vasodilatation and inotropic actions induced by the HNO donor, Angeli’s salt (sodium trioxodinitrate), are sGC-dependent and sGC-independent, respectively.

## Materials and methods

Haemodynamic responses to bolus doses of Angeli’s salt (10pmol - 10μmol), alone and in the presence of selective scavengers of HNO (L-cysteine, 4mM) or NO (hydroxocobalamin HXC, 50µM) or selective inhibitors of sGC (1H-[1,2,4]oxadiazolo[4,3-a]quinoxalin-1-one ODQ, 10μM), calcitonin gene-related peptide (CGRP) receptors (CGRP_8-37_, 0.1μM) or voltage-dependent potassium channels (4-aminopyridine 4-AP, 1mM) were determined in isolated adult male rat hearts.

## Results

Angeli’s salt elicited concomitant, potent dose-dependent increases in coronary flow and LV systolic and diastolic function. Both L-cysteine and ODQ caused a rightward shift in the dose-response curve of each of these effects, implicating HNO and sGC in both the vasodilator and inotropic actions of Angeli’s salt. In contrast, neither HXC, CGRP_8-37_ nor 4-AP affected Angeli’s salt actions.

## Conclusion

These data suggest that each of the vasodilator, inotropic and lusitropic actions of Angeli’s salt are mediated by L-cysteine-sensitive, HNO/sGC-dependent mechanisms. Our findings represent the first evidence that sGC specifically contributes a significant component of the inotropic and lusitropic actions of an HNO donor in the intact heart. Thus, HNO acutely enhances LV contractile function and LV relaxation, whilst concomitantly unloading the heart, potentially favourable properties for the failing heart.

